# Biological characteristics and fungicide sensitivity of *Pyricularia variabilis*

**DOI:** 10.1515/biol-2021-0095

**Published:** 2021-09-06

**Authors:** Yiming Zhang, Tingguan Wu, Yonghong He

**Affiliations:** Huzhou Vocational & Technical College, Huzhou, Zhejiang 313000, People’s Republic of China; Horticultural Research Institute of Jiangxi Academy of Agricultural Sciences, Nanchang, Jiangxi 330200, People’s Republic of China; College of Plant Protection, Yunnan Agricultural University, Kunming, Yunnan 650201, People’s Republic of China

**Keywords:** *Amomum tsao-ko*, leaf blast, mycelial growth, *Pyricularia variabilis*, plant disease

## Abstract

In recent years, the pathogen that causes leaf blast on *Amomum tsao-ko* repeatedly infected the plants in a large area of Luchun County, Honghe Prefecture, Yunnan Province, China. The disease is caused by the pathogen *Pyricularia variabilis*. The effects of light, temperature, pH, carbon, and nitrogen sources on the growth of the pathogen were determined, and its sensitivity to six fungicides was determined using the mycelial growth rate method. The optimal conditions for mycelial growth were as follows: temperature: 20–25°C; carbon source: maltose, nitrogen source beef extract, media corn flour, and potato dextrose agar. The mycelia could grow under four types of light conditions: 24 h light, 24 h dark, 12 h light/12 h dark, and 16 h light/8 h dark. In addition, Propiconazole was the most effective inhibitor, with an EC_50_ value of 0.030 μg/mL, and prochloraz was the second most effective, with an EC_50_ value of 0.076 μg/mL. It is suggested that the two fungicides should be alternated when used in production. Carbendazim and chlorothalonil were ineffective in inhibiting the fungus, with EC_50_ values of 6.137 and 3.765 μg/mL, respectively.

## 1 Introduction

*Amomum tsao-ko* is a perennial evergreen economic plant in the *Myristica* genus in the Zingiberaceae family [1]. It usually grows in shady and humid forests in tropical and subtropical zones. Its growth requires strict ecological conditions. The plant is a common Chinese herbal medicine and an important seasoning ingredient in various dishes [2]. *Amomum tsao-ko* is primarily cultivated in Yunnan, China. In recent years, 80% of the plants in the Honghe Prefecture of Yunnan Province have been damaged by leaf blasts [3]. The specific symptoms of the leaf blast on *Amomum tsao-ko* are water spots on the leaves, which gradually expand into rhombic, fusiform and spindle shapes. The middle part of the lesion is gray and white, which is surrounded by brown necrotizing tissue, and the edge is faded green. When the environmental humidity is high, a layer of brown mold appears on the surface of the lesion, and then the lesions expand and coalesce, resulting in leaf wilting and plant death [4]. The pathogen was isolated and identified in 2015 [4]. *P. variabilis*, the causal agent of the leaf blast of *Amomum tsao-ko*, belongs to Deuteromycotina, Hyphomycetes, Hyphomycetales, Moniliaceae, *Pyricularia* Sacc. according to the system of Ainsworth＆Alexopoulos. The genus *Pyricularia* has a wide host range and is best known for the pathogen *P. oryzae*, the causal agent of rice blast, one of the most devastating diseases of rice [5]. *Pyricularia* can also infect *Cauda equisetifolia, Zizania latifolia*, *Digitaria tomentosa*, corn, and oats, producing leaf spots similar to those of rice blast. In 2003, Busshan first found *P. variabilis* on *Amomum siamense* leaves [6], and in 2017, he identified the pathogen using molecular and morphological techniques [7]. No data on the susceptibility of *Pyricularia variabilis* to fungicides and determination of its biological characteristics has been reported until now. In recent years, owing to the frequent use of commonly used broad-spectrum fungicides by the local growers, the resistance of fungal pathogens has become an enormous problem. The fungi that are resistant to fungicides only comprise a small part of the total number of pathogens at the beginning of the use of these compounds, but the proportion of resistant individuals may increase with the use of fungicides over a long period. Thus, the ability of fungicides to control the pathogens decreased, and the prevalence of diseases increased yearly [8]. Chemical fungicides are still the main means to control diseases caused by the subfamily Deuteromyces that are found all over the world [9]. To provide a valuable theoretical and practical basis for the prevention and control of leaf blast pathogen and to limit the development in the pathogen, a toxicity test of *P. variabilis* was conducted in the laboratory.

Carbendazim is a benzimidazole, whereas difenoconazole and propiconazole are triazoles. Moreover, chlorothalonil is substituted benzene, whereas prochloraz is an imidazole, and azoxystrobin is a kind of strobilurin fungicide. Benzimidazoles act by binding to microtubules and stopping hyphal growth. They also bind to the spindle microtubules and block nuclear division. The most popular fungicide in this class is carbendazim [10], which is effectively used to inhibit the growth of fungi from the phylum of Deuteromyces. Triazoles interfere with ergosterol biosynthesis. They specifically bind to the enzyme 14α-demethylase encoded by the CYP51 gene [11], inhibiting its activity, stopping the synthesis of ergosterol in the cell membrane, and changing the permeability of the cell membrane, causing the loss of important substances in the cell and eventually resulting in the death of the fungus.

Owing to their special physical and chemical properties, strobilurin fungicides have become popular throughout the world in recent years to control fungal diseases in agriculture. They are widely used in the United States as effective fungicidal agents in the cultivation of crops. Similarly, the UK has experienced an increase in the use of strobilurin fungicides over the previous decade [12], and the strobilurins ranked first in global sales among fungicides in 2013 [13]. The molecular target of strobilurin fungicides is the mitochondrial respiratory complex III, which is an integral membrane protein complex that couples the electron transfer from quinol to cytochrome c1 with proton translocation across the membrane. Strobilurins act by binding to the Q0 site of complex III to block the electron transfer between cytochrome b and cytochrome c1, and thereby cause a deficiency in ATP synthase to inhibit cellular respiration in eukaryotes [14].

The average pH value of the soil in the sample collection area is 5.5, and the annual average temperature ranges from 20 to 30°C. The disease in the shaded areas is relatively serious. Researchers infer that the acidic environment, the growth temperature (20–30°C), and the weak light condition are very favorable for the growth of *P. variabilis*. In order to provide relevant data for additional study on its pathogenesis, breeding for disease resistance, and testing the researchers’ inference, research on the biological characteristics of *P. variabilis* has been conducted in this study. We analyzed the effects of various medium formulations, temperature, pH, light, carbon, and nitrogen sources on the growth of the pathogen along with the fungicide sensitivity of *P. variabilis*.

## 2 Materials and methods

### 2.1 Isolation of the pathogen

In 2018, a total of seven monoconidial isolates of *P. variabilis* were collected from *Amomum tsao-ko* diseased leaves from seven different *Amomum tsao-ko* orchards in Luchun County (E: 102°17′, N: 22°50′), Honghe Prefecture, Yunnan Province, China. One of seven isolates was tested in this study.

After the isolation of a pure culture, they were stored at 4°C in potato dextrose agar (PDA) media. Before the experiment was conducted, the strains were transferred to PDA media at 27°C for five days until the culture had fully expanded.

### 2.2 Production of culture media

The PDA medium was prepared by selecting 200 g of peeled potatoes and cutting them into small pieces. The pieces were boiled in water, filtered with eight layers of gauze, and heated. Then, 20 g of agar was added, heated, and stirred. After the agar had dissolved, 20 g of glucose was added, stirred, cooled slightly, and water was added to bring the volume to 1,000 mL. The medium was sterilized and poured into each dish (⌀ = 90 mm), 15 mL per dish, which was used for experiments.

The potato saccharose agar (PSA) medium was prepared in the same manner as PDA with the exception that sucrose was added instead of glucose.

The V8 juice culture medium was prepared with 200 mL of V8 and 20 g of agar, which was heated and mixed well. Three grams of 3G CaCO_3_ was added after the agar had dissolved. Water was added to bring the volume to 1,000 mL after the solution had been mixed and cooled. The medium was sterilized and poured into each dish (⌀ = 90 mm), 15 mL per dish, which was used for experiments.

The corn flour culture medium was prepared by heating 500 mL of water to 70°C and then adding 40 g of corn flour. The solution was incubated at 60°C for approximately 1 h, filtered with gauze, and brought to a volume of 500 mL with water. A separate solution was prepared by adding agar to 500 mL of water and heating it so that the agar melted slowly. The solution was filtered and brought to 500 mL by the addition of water. The two solutions are mixed and sterilized and then poured into each dish (⌀ = 90 mm), 15 mL per dish, which was used for experiments.

The preparation of the Czapek medium involved the heating of 20 g of agar and 1,000 mL distilled water. After the agar was dissolved, 2 g of sodium nitrate, 1 g of dipotassium hydrogen phosphate, 0.5 g of KCl, 0.5 g of magnesium sulfate heptahydrate, 0.01 g of ferrous sulfate heptahydrate, and 20 g of sucrose were added. The mixture was stirred evenly, cooled slightly, and then subpacked into conical bottles for sterilization. After sterilization, the medium was poured into each dish (⌀ = 90 mm), 15 mL per dish, which was used for experiments.

Thirty grams of oatmeal was added to 1,000 mL of water to prepare the oatmeal culture medium. The solution was boiled in a water bath for 1 h and filtered with gauze. Twenty grams of agar was added to 1,000 mL of water, which was subsequently sterilized. After sterilization, the medium was poured into each dish (⌀ = 90 mm), 15 mL per dish, which was used for experiments.

The *Amomum tsao-ko* stalk decoction medium was prepared in the same manner as the PDA, except that the fresh *Amomum tsao-ko* stalk was added instead of potatoes. After sterilization, the medium was poured into each dish (⌀ = 90 mm), 15 mL per dish, which was used for experiments.

The Richard medium was prepared by mixing and heating 20 g of agar and 1,000 mL distilled water. After the agar had dissolved, 10 g of potassium nitrate, 5 g of potassium dihydrogen phosphate, 2.5 g of magnesium sulfate heptahydrate, 0.02 g of ferric chloride, and 50 g of sucrose were added. The mixture was stirred evenly, cooled slightly, and then subpacked into conical bottles for sterilization. After sterilization, the medium was poured into each dish (⌀ = 90 mm), 15 mL per dish, which was used for experiments.

The water agar medium was prepared by mixing 20 g of agar and 1,000 mL of distilled water and heated. After the agar was dissolved, the medium was packed into conical bottles for sterilization. After sterilization, the medium was poured into each dish (⌀ = 90 mm), 15 mL per dish, which was used for experiments.

### 2.3 Biological characteristics of *P. variabilis*

#### 2.3.1 Effects of different media formulations on the growth of *P. variabilis*

*P. variabilis* mycelia obtained from a PDA slant culture were transferred to PDA plates and grown for 7 days in a 25°C incubator. A total of 45 mycelial plugs with a diameter of 5 mm were obtained from the plates using a hole puncher. The test plugs were placed in the center of different types of media described above. One plug was inoculated per Petri dish (⌀ = 90 mm) and grown for 7 days in a 25°C incubator. The diameter of the colonies was determined and recorded using the cross method. Five replicates per treatment were conducted.

#### 2.3.2 Effect of different temperatures on the growth of *P. variabilis*

Mycelial plugs (⌀ = 5 mm) of *P. variabilis* cultured on PDA media for 7 days were placed in the center of a PDA Petri dish. Each dish (⌀ = 90 mm) was inoculated with one plug. The dishes were placed in an incubator at 5, 10, 15, 20, 25, 30, and 35°C for cultivation and the colony diameter was measured and recorded 7 days later using the cross method. Five replicates were conducted per treatment.

#### 2.3.3 Effect of different pH values on the growth of *P. variabilis*

The pH value of PDA was adjusted by 1 mol/L hydrochloric acid and 1 mol/L sodium hydroxide using a pH meter under aseptic conditions. The gradient of media with pH of 3, 4, 5, 6, 7, 8, 9, 10, and 11 was established. Mycelial plugs (⌀ = 5 mm) that had grown on PDA media for 7 days were inoculated on PDA dishes with different pH gradients. Each dish (⌀ = 90 mm) was inoculated with one plug. Five replicates were conducted per pH value. The cultures were grown and measured as described above.

#### 2.3.4 Effect of different light conditions on the growth of *P. variabilis*

Mycelial plugs (⌀ = 5 mm) that had grown on PDA for 7 days were inoculated on PDA dishes and incubated at different light condition gradients, including 16:8 natural light, 12:12 light/dark alternation, 24 h full darkness, and 24 h full light. Each dish (⌀ = 90 mm) was inoculated with one plug. Five replicates were conducted for each treatment, and the treatments were evaluated as described above.

#### 2.3.5 Effect of different carbon sources on the growth of *P. variabilis*

The Czapek medium was used as the basic medium; different carbon sources were prepared by replacing sucrose with the same weight (in g) of alternate carbon sources, such as malt dust, starch, α-lactose, glucose, and mannitol. Media that lacked the carbon source was used as a blank control. Mycelial plugs (⌀ = 5 mm) were grown and evaluated as described above. Each dish (⌀ = 90 mm) was inoculated with one plug.

#### 2.3.6 Effect of different nitrogen sources on the growth of *P. variabilis*

Using the Czapek medium as the basic medium, different nitrogen sources were prepared by replacing sodium nitrate with the same amount of alternate nitrogen sources, such as beef paste, ammonium chloride, potassium nitrate, glycine, ammonium sulfate, urea, and peptone.

The medium without a nitrogen source was used as a blank control. Mycelial plugs (⌀ = 5 mm) were grown and evaluated as described above. Each dish (⌀ = 90 mm) was inoculated with one plug.

#### 2.3.7 Data analysis

SPSS 19.0 (IBM, Inc., Armonk, NY, USA) was used to calculate the mean colony diameter, and Duncan’s new complex range method was used to analyze the significant difference in Duncan’s multiple range test (DMRT).

### 2.4 Evaluating the sensitivity of *P. variabilis* to six fungicides

#### 2.4.1 Test materials

##### 2.4.1.1 Fungicides

The fungicides tested included propiconazole 95% technical (Shanghai Forever Chemical Co., Ltd., Shanghai, China), azoxystrobin 98.5% technical (Jiangyin Suli Chemical Co., Ltd.), chlorothalonil 98% technical (Shandong Huayang Pesticide Fertilizer Chemical Co., Ltd.), difenoconazole 98% technical (Hangzhou Yulong Agrochemical Co., Ltd.), prochloraz 97% technical (Hubei Shenglong Chemical Co., Ltd.), and carbendazim 98% technical (Shanghai Linkong Chemical Trade Co., Ltd.). Dimethyl sulfoxide (DMSO) was used as a solvent and mixed with the technical drug to make stock solutions.

#### 2.4.2 Test methods

The mycelial growth rate was monitored in this experiment [15]. Stock solutions were prepared in DMSO to a concentration of 100 µg/mL [16]. Aliquots were added to approximately 15 mL of 45°C PDA per plate. The Petri dish containing (1) only DMSO served as a negative control, (2) only sterile water served as blank control, and (3) fungicide and DMSO served as a positive control [17]. The colony diameters were measured in a cross pattern. The original plug diameter was subtracted, and the mean of colony diameters was calculated. Each concentration was tested in triplicate. The rate of inhibition of *P. variabilis* to propiconazole was determined by transferring mycelial plugs (⌀ = 5 mm) from 5-day-old (27°C) *P. variabilis* colonies placed in the center of PDA media amended with 200, 100, 50, 20, 10, 5, 2.5, 2, 1, 0.5, 0.2, 0.1, 0.05, 0.02, 0.01, 0.001, 0.005, and 0.0001 µg/mL of propiconazole. The data for inhibition rates were expressed as CGI = ((MCDc–MCDf)/MCDc) × 100 [18], where MCDc is the mean colony diameter of the negative control and MCDf is the mean colony diameter of the positive control. Eight PDA concentration grades of propiconazole with inhibition rates of 5–95% were selected and determined using the software. The fungicidal PDA concentration grades for the other fungicides were determined in a similar manner. SPSS 19.0 was used to calculate the average values of the colony diameter and rate of inhibition. DMRT was used to analyze the significant differences between group means using SPSS 19.0. Moreover, the probit analysis function of SPSS 19.0 was used to determine the effective concentration at which the mycelial growth was inhibited by 50% (EC_50_), the correlation coefficient, and the regression equations of the toxicity of six different fungicides to *P. variabilis*. SPSS19.0 software for data processing was mainly to take logarithm (*x*) of the fungicide concentration obtained in the experiment and convert the inhibition rate into probability value (*y*) so as to draw the toxicity regression equation. The closer the value of correlation coefficient “*r*” calculated by software is to 1, the stronger the linear correlation between logarithmic concentration and probability value of the fungicide (Table 1).

**Table 1:** Initial concentration and concentrations of the fungicides in PDA

Fungicides	Initial concentration (μg/mL)	Concentrations in PDA (μg/mL)
Propiconazole	100	0.0005, 0.001, 0.01, 0.02, 0.1, 0.5, 1, 5
Azoxystrobin	100	0.01, 0.05, 0.1, 0.2, 0.5, 1, 2, 5
Chlorothalonil	100	0.05, 0.1, 0.5, 1, 2.5, 10, 20, 50
Difenoconazole	100	0.5, 1, 2, 2.5, 5, 10, 15, 30
Prochloraz	100	0.01, 0.02, 0.05, 0.1, 0.2, 0.5, 1, 2
Carbendazim	100	0.1, 0.5, 1, 5, 10, 20, 50, 100

## 3 Results

### 3.1 Biological characteristics of the pathogen

#### 3.1.1 Effects of different media and light conditions on the mycelial growth of *P. variabilis*

It can be clearly observed in Table 2 that the pathogen can grow on nine different types of media. There was a significant difference in the colony growth diameter after 7 days of cultivation at 25°C. *P. variabilis* grew most quickly on corn flour media, with the colony diameter reaching 7.09 cm. In contrast, the fungus grew slowly on water agar and oatmeal media.

**Table 2:** The growth of colony on different types of media

Medium type	Mean colony diameter (cm)	Uniformity	Mycelial growth vigor
Corn flour medium	7.09 ± 0.35a	Regular	＋＋＋
PDA medium	6.34 ± 0.31b	Regular	＋＋＋
PSA medium	5.86 ± 0.17c	Regular	＋＋＋
V8 juice culture	5.76 ± 0.21cd	Regular	＋＋＋
Czapek medium	5.50 ± 0.23cd	Regular	＋
Richard medium	5.39 ± 0.24d	Regular	＋＋
*Amomum tsao-ko* stalk medium	4.91 ± 0.25e	Regular	＋＋
Oatmeal medium	3.91 ± 0.30f	Irregular	＋
Water agar medium	3.19 ± 0.14g	Irregular	＋

(1) +++ indicates that the mycelia grow vigorously and densely; ++ indicates that the mycelia grow ordinarily; and + indicates that the mycelia grow weakly and sparsely.

(2) Uniformity indicates the uniformity of colony edge.

(3) Note: The small letters in the figure are of 5% significance.

**Figure 1:**
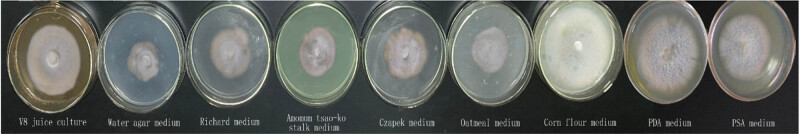
The morphology, growth vigor, and uniformity of mycelium under different medium conditions.

**Figure 2:**
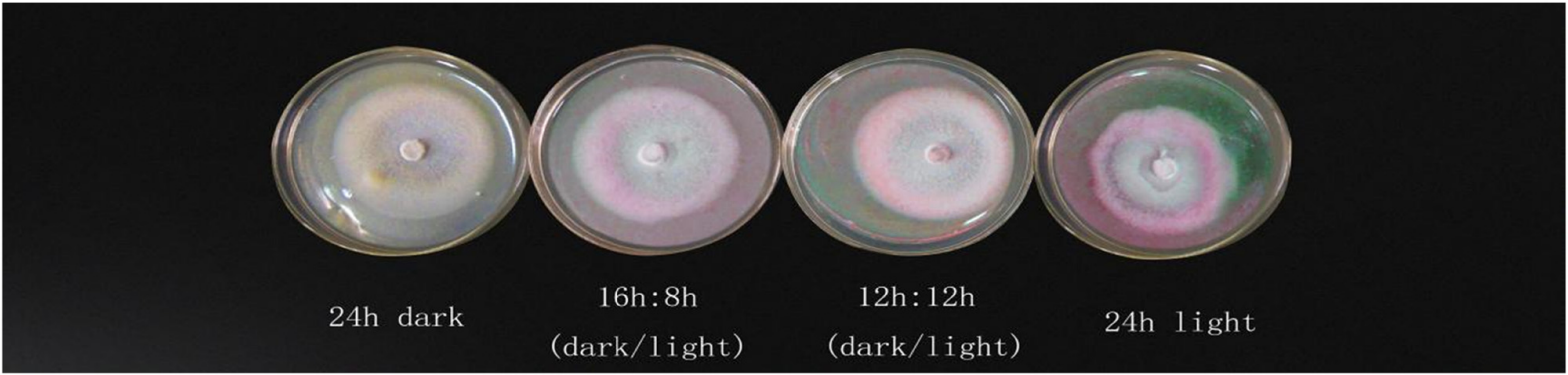
The morphology, growth vigor, and uniformity of mycelium under different light conditions.

It can be also clearly observed in Table 3 that the average diameter of the colony was 6.32 cm under 24 h of darkness and 5.82 cm under 24 h full light. Thus, darkness was more suitable for the growth of mycelia. There are differences in morphology, growth vigor, and uniformity of the mycelium under different medium conditions and light conditions, as seen from Figures 1 and 2.

**Table 3:** The growth of colony on PDA media under different light conditions

Light condition	Mean colony diameter	Uniformity	Mycelial growth vigor
24 h dark	6.32 ± 0.15a	Regular	＋＋＋
16:8 (dark/light)	6.17 ± 0.18ab	Regular	＋＋＋
12:12 (dark/light)	6.02 ± 0.22ab	Regular	＋＋＋
24 h light	5.82 ± 0.25b	Regular	＋＋＋

(1) +++ indicates that the mycelia grow vigorously and densely; ++ indicates that the mycelia grow ordinarily; and + indicates that the mycelia grow weakly and sparsely.

(2) Uniformity indicates the uniformity of colony edge.

(3) Note: The small letters in the figure are of 5% significance.

#### 3.1.2 Effects of different carbon and nitrogen sources on the mycelial growth of *P. variabilis*

**Figure 3:**
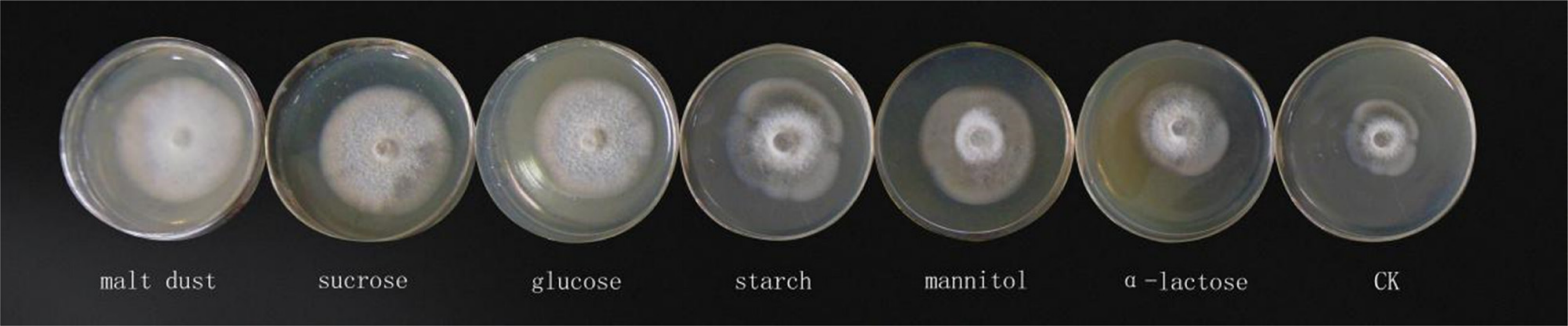
The morphology, growth vigor, and uniformity of mycelium under different carbon sources.

**Figure 4:**
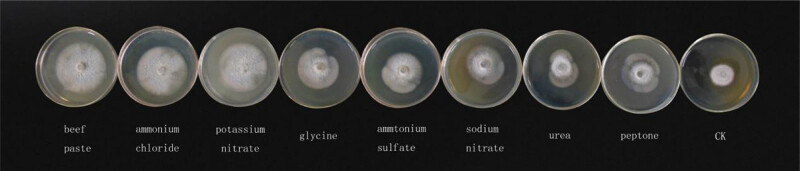
The morphology, growth vigor, and uniformity of mycelium under different nitrogen sources.

It is apparent from Tables 4 and 5 that the pathogen can grow when different carbon and nitrogen sources are supplied. The fungus grew most quickly and had the largest colony diameter when grown on maltose as a carbon source and the slowest growth was observed on α-lactose. There are significant differences between these two data. When the beef extract was used as the nitrogen source, the pathogen grew most quickly; the colony diameter was the largest. The slowest growth was observed on the peptone. The slowest data are significantly different from the highest. There are significant differences in morphology, growth vigor, and uniformity of mycelium when different carbon and nitrogen sources were used, as seen from Figures 3 and 4.

**Table 4:** The growth of colony on different carbon sources media

Carbon sources	Mean colony diameter (cm)	Uniformity	Mycelial growth vigor
Malt dust	6.19 ± 0.22a	Regular	＋＋＋
Sucrose	5.80 ± 0.23b	Regular	＋＋＋
Glucose	5.48 ± 0.25bc	Regular	＋＋＋
Starch	5.39 ± 0.19c	Regular	＋＋
Mannitol	4.46 ± 0.18d	Irregular	＋＋
α-Lactose	3.83 ± 0.12e	Regular	＋
CK	3.22 ± 0.25f	Irregular	＋

(1) +++ indicates that the mycelia grow vigorously and densely; ++ indicates that the mycelia grow ordinarily; and + indicates that the mycelia grow weakly and sparsely.

(2) Uniformity indicates the uniformity of colony edge.

(3) Note: The small letters in the figure are of 5% significance.

**Table 5:** The growth of colony on media with different nitrogen sources

Nitrogen sources	Mean colony diameter (cm)	Uniformity	Mycelial growth vigor
Beef paste	5.82 ± 0.25a	Regularr	＋＋＋
Ammonium chloride	5.54 ± 0.25ab	Regular	＋＋＋
Potassium nitrate	5.31 ± 0.21b	Regular	＋＋＋
Glycine	4.86 ± 0.22c	Regular	＋＋
Ammonium sulfate	4.75 ± 0.19c	Regular	＋＋
Sodium nitrate	4.60 ± 0.19cd	Regular	＋
Urea	4.36 ± 0.34de	Irregular	＋＋
Peptone	4.12 ± 0.14e	Irregular	＋
CK	3.69 ± 0.21f	Irregular	＋＋

(1) +++ indicates that the mycelia grow vigorously and densely; ++ indicates that the mycelia grow ordinarily; and + indicates that the mycelia grow weakly and sparsely.

(2) Uniformity indicates the uniformity of colony edge.

(3) Note: The small letters in the figure are of 5% significance.

#### 3.1.3 Effects of different pH values and temperatures on the mycelial growth of *P. variabilis*

**Figure 5:**
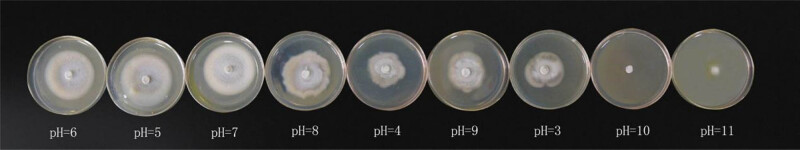
The morphology, growth vigor, and uniformity of mycelium under different pH values.

**Figure 6:**
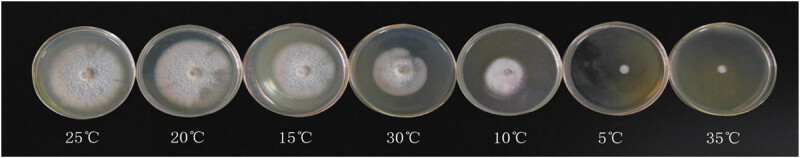
The morphology, growth vigor, and uniformity of mycelium under different temperatures.

As shown in Tables 6 and 7, the pathogen can grow in the temperature range of 10–30°C, and 20–25°C is the most suitable temperature range for its growth. The colony diameter was the largest when the temperature was 25°C and reached 7.45 cm. At 10°C, the colonies grew very slowly and the colony radius was only 2.92 cm. The mycelia could not grow at 5–35°C. The suitable pH range for mycelial growth was 5.0–7.0, and the mycelia grew most quickly at pH 6.0. Thus, the pathogen is better adapted to an acidic environment. It can be seen from Figures 5 and 6 that the morphology, growth vigor, and uniformity of mycelium are different under different pH values and temperatures.

**Table 6:** The growth of colony on PDA media under different temperatures

Test temperature (°C)	Mean colony diameter (cm)	Uniformity	Mycelial growth vigor
25	7.45 ± 0.20a	Regular	＋＋＋
20	7.23 ± 0.21a	Regular	＋＋＋
15	5.12 ± 0.25b	Regular	＋＋＋
30	4.66 ± 0.22c	Regular	＋＋
10	2.92 ± 0.4d	Regular	＋＋
5	0.00 ± 0.00e	Irregular	＋
35	0.00 ± 0.00e	Irregular	＋

(1) +++ indicates that the mycelia grow vigorously and densely; ++ indicates that the mycelia grow ordinarily; and + indicates that the mycelia grow weakly and sparsely.

(2) Uniformity indicates the uniformity of colony edge.

(3) Note: The small letters in the figure are of 5% significance.

**Table 7:** The growth of colony on PDA media under different pH values

pH value	Mean colony diameter (cm)	Uniformity	Mycelial growth vigor
6	6.39 ± 0.17a	Regular	＋＋＋
5	5.94 ± 0.19b	Regular	＋＋＋
7	5.83 ± 0.33bc	Regular	＋＋＋
8	5.47 ± 0.31cd	Irregular	＋
4	5.39 ± 0.26d	Irregular	＋＋
9	3.76 ± 0.17e	Irregular	＋
3	2.83 ± 0.25f	Irregular	＋
10	2.36 ± 0.14g	Irregular	＋
11	0.00 ± 0.00h	Irregular	＋

(1) +++ indicates that the mycelia grow vigorously and densely; ++ indicates that the mycelia grow ordinarily; and + indicates that the mycelia grow weakly and sparsely.

(2) Uniformity indicates the uniformity of colony edge.

(3) Note: The small letters in the figure are of 5% significance.

### 3.2 Evaluation of toxicity of six fungicides to *P. variabilis*

#### 3.2.1 The inhibitory effect of six fungicides on the growth of *P. variabilis*

The inhibitory effect of six fungicides tested on the mycelial growth of *P. variabilis* is shown in Table 8. The same fungicide had different inhibitory effects at varying concentrations in the plate, and the rates of inhibition by propiconazole, azoxystrobin, chlorothalonil, difenoconazole, prochloraz, and carbendazim were 13.15–94.62%, 11.35–94.42%, 5.38–89.64%, 15.14–94.22%, 23.51–90.24%, and 8.37–93.43%, respectively.

**Table 8:** The toxicity of six types of single fungicides on *Pyricularia variabilis*

Fungicide	PDA concentrations (μg/mL)	Colony diameter (cm)	Inhibition rates (%)	Toxicity regression equations	Correlation coefficient (*r*)	EC_50_ (μg/mL)	95% CL/μg/mL
Propiconazole	0.0005	4.36 ± 0.07b	13.15	*y* = 0.589*x* + 0.96	0.984	0.030	0.020–0.044
	0.001	3.73 ± 0.09c	25.70				
	0.01	3.12 ± 0.12d	37.85				
	0.02	2.84 ± 0.04e	43.43				
	0.1	2.1 ± 0.18f	58.17				
	0.5	1.43 ± 0.11g	71.51				
	1	0.83 ± 0.22h	83.47				
	5	0.27 ± 0.14i	94.62				
	CK	5.02 ± 0.16a	—				
Azoxystrobin	0.01	4.45 ± 0.18b	11.35	*y* = 1.09*x* + 0.618	0.981	0.271	0.185–0.393
	0.05	3.93 ± 0.17c	21.71				
	0.1	3.60 ± 0.10d	28.29				
	0.2	3.25 ± 0.17e	35.26				
	0.5	2.34 ± 0.30f	58.39				
	1	1.33 ± 0.13g	73.51				
	2	0.55 ± 0.12h	89.04				
	5	0.23 ± 0.06i	94.42				
	CK	5.02 ± 0.16a	—				
Chlorothalonil	0.05	4.75 ± 0.18b	5.38	*y* = 0.82*x* − 0.472	0.979	3.765	2.337–6.381
	0.1	4.40 ± 0.10c	12.35				
	0.5	3.94 ± 0.15d	21.51				
	1	3.18 ± 0.08e	36.65				
	2.5	2.83 ± 0.14f	43.63				
	10	2.39 ± 0.16g	52.39				
	20	1.44 ± 0.12h	71.31				
	50	0.52 ± 0.12i	89.64				
	CK	5.02 ± 0.16a	—				
Difenoconazole	0.5	4.26 ± 0.16b	15.14	*y* = 1.283*x* − 0.611	0.995	2.991	2.496–3.561
	1	3.84 ± 0.17c	23.51				
	2	2.95 ± 0.19d	41.24				
	2.5	2.39 ± 0.20e	52.39				
	5	1.83 ± 0.14f	63.55				
	10	1.40 ± 0.16g	72.11				
	20	0.78 ± 0.08h	84.46				
	50	0.29 ± 0.13i	94.22				
	CK	5.02 ± 0.16a	—				
Prochloraz	0.01	3.84 ± 0.11b	23.51	*y* = 0.881*x* + 0.986	0.998	0.076	0.058–0.098
	0.02	3.49 ± 0.15c	30.48				
	0.05	2.91 ± 0.15d	42.03				
	0.1	2.31 ± 0.22e	53.98				
	0.2	1.89 ± 0.16f	62.35				
	0.5	1.11 ± 0.23g	77.89				
	1	0.83 ± 0.03h	83.47				
	2	0.49 ± 0.08i	90.24				
	CK	5.02 ± 0.16a	—				
Carbendazim	0.1	4.6 ± 0.20b	8.37	*y* = 0.877*x* − 0.691	0.976	6.137	3.947–9.594
	0.5	4.09 ± 0.13c	18.53				
	1	3.72 ± 0.10d	25.90				
	5	3.05 ± 0.15e	39.24				
	10	2.3 ± 0.08f	54.18				
	20	1.94 ± 0.17g	61.35				
	50	1.00 ± 0.25h	80.08				
	100	0.33 ± 0.18i	93.43				
	CK	5.02 ± 0.16a	—				

Note: The colony diameter and rates of inhibition are the results of 72 h culture of experimental colony. The small letters after the average diameter indicate that the difference is significant (*P* ≤ 0.05) by Duncan’s new multiple range difference method (DMRT). “*r*” indicates the correlation coefficient.

#### 3.2.2 The sensitivity of *P. variabilis* to six different fungicides

Among the fungicides tested, propiconazole was the most effective inhibitor (Table 8). It was the most toxic, with an EC_50_ value of 0.030 μg/mL. These data suggest that propiconazole would be the first choice of the fungicide to control *P. variabilis*, followed by prochloraz and azoxystrobin, with EC_50_ values of 0.076 and 0.271 μg/mL, respectively. These compounds could be used as alternatives to control *P. variabilis*. Among the six fungicides, carbendazim had the highest EC_50_ value (6.137 μg/mL) to *P. variabilis*, which indicated that carbendazim was the least toxic and the worst inhibitor of *P. variabilis*. The EC_50_ value of chlorothalonil was similar to carbendazim, which was 3.765 μg/mL.

## 4 Discussion

This is the first report on the study of biological characteristics and fungicide sensitivity of *Pyricularia variabilis*. In this paper, the biological characteristics of *P. variabilis* were studied to provide a theoretical basis to clarify the relationship between the growth of pathogen and environmental conditions. There were significant differences in the growth of the pathogen on different media. The pathogen grew well with dense colonies on the corn flour, whereas slow and sparse growth was observed on the water agar. But, we only evaluated the suitability of the nine media for growth, and more studies are still needed to determine the optimum environmental conditions for the growth of *P. variabilis on* different media.

The optimal pH range for the mycelial growth of *P. variabilis* was 5.0–7.0, indicating that the pathogen prefers to grow well in acidic environments. Under different pH conditions, the colony morphology and growth vigor changed significantly, which was consistent with the article on the biological characteristics of *Pyricularia grisea* isolated from the leaves of rice [19].

The optimal range of temperature for pathogen growth on PDA was 20–25°C, slightly different from the previous report of optimal growth at a temperature of 28°C of *Pyricularia grisea* isolated from the leaves of rice [19]. The average temperature of the sample collection area (E: 102°17′, N: 22°50′) is 23°C in spring and the occurrence of diseases is also concentrated in this season. This is consistent with the data of the ideal range of temperatures for pathogen growth on PDA obtained in this experiment. These results contribute to the understanding of the etiology and epidemiology of the disease. In the light of our experiment, the results showed that the growth of *P. variabilis* was not very sensitive to light. So, maybe it is ineffective to take measures of shading and avoiding light for controlling the growth and spread of *P. variabilis.*

*P. variabilis* is a type of pathogen that can use multiple types of carbon and nitrogen as sources for its nutrition. Compared with the blank control without carbon and nitrogen sources, growth differed significantly indicating that the demand for carbon and nitrogen sources is very important. The farmers should control the amount of fertilizers that contain carbon and nitrogen sources and do not overuse fertilizers. After planting of *Amomum tsao-ko*, topdressing should be carried out 2–3 times. The first topdressing should be completed in March, which can promote the growth of grass and fruit. Green manure, compost, or stable manure can be selected. The second topdressing is usually carried out at the end of August, which can enhance the cold resistance of seedlings and make the grass and fruit blossom and bear fruit as soon as possible. The amount of plant ash applied per mu is 150 kg and the amount of burnt soil is 2 t. The third topdressing is mostly carried out around the middle of April to promote pollen development, mainly by spraying 0.3% potassium dihydrogen phosphate and 0.01% boric acid mixture.

There are no reports that document the effect of fungicides on *P. variabilis.* This study aims to provide information about the sensitivity of *P. variabilis* to different types of fungicides. The EC_50_ can be used as a reliable standard to measure the effect of fungicides. The smaller the value, the better the effect. The results of this experiment showed that propiconazole showed a high inhibitory effect on *P. variabilis,* which was consistent with the article on the fungicides against *Pyricularia setariae* isolated from the leaf blast in foxtail millet [20].

The use of prochloraz and azoxystrobin can be alternated to delay the development of resistance to fungicides. More field study is required to choose effective fungicides [21]. The closer the correlation coefficient (“*r*”) is to 1, the higher the degree of linear correlation between variables (“Logarithm of concentration” and “probit”), and the higher the fitting degree of the straight line of the toxicity regression equation [22]. A limited selection of fungicides was used in this experiment, and it cannot be ruled out that other environmentally friendly, inexpensive, and effective fungicides could also inhibit the growth of *P. variabilis*. The EC_50_ value is only the reference value for a laboratory assay, and the relevant effective chemicals should be screened in field trials to explore the time of application and method to optimize control. The management of this disease requires an integrated approach, among which cultural practices must be emphasized to delay the development of resistance and maintain efficacy that directly impacts yield [23]. In addition, the cost, stability of dosage form, field climate, and environmental factors should be considered in the actual field application.
